# Time-course of cortical networks involved in working memory

**DOI:** 10.3389/fnhum.2014.00004

**Published:** 2014-01-29

**Authors:** Phan Luu, Daniel M. Caggiano, Alexandra Geyer, Jenn Lewis, Joseph Cohn, Don M. Tucker

**Affiliations:** ^1^Electrical Geodesics, Inc., EugeneOR, USA; ^2^Department of Psychology, University of Oregon, EugeneOR, USA; ^3^Aptima, Inc., WoburnMA, USA; ^4^Office of Naval Research, ArlingtonVA, USA

**Keywords:** dense-array EEG, working memory, attention, temporal parietal junction, frontal lobe

## Abstract

Working memory (WM) is one of the most studied cognitive constructs. Although many neuroimaging studies have identified brain networks involved in WM, the time course of these networks remains unclear. In this paper we use dense-array electroencephalography (dEEG) to capture neural signals during performance of a standard WM task, the *n*-back task, and a blend of principal components analysis and independent components analysis (PCA/ICA) to statistically identify networks of WM and their time courses. Results reveal a visual cortex centric network, that also includes the posterior cingulate cortex, that is active prior to stimulus onset and that appears to reflect anticipatory, attention-related processes. After stimulus onset, the ventromedial prefrontal cortex, lateral prefrontal prefrontal cortex, and temporal poles become associated with the prestimulus network. This second network appears to reflect executive control processes. Following activation of the second network, the cortices of the temporo-parietal junction with the temporal lobe structures seen in the first and second networks re-engage. This third network appears to reflect activity of the ventral attention network involved in control of attentional reorientation. The results point to important temporal features of network dynamics that integrate multiple subsystems of the ventral attention network with the default mode network in the performance of working memory tasks.

## INTRODUCTION

An essential capacity for cognition and human performance is working memory (WM), the ability to hold information in store as it is manipulated through various cognitive transformations. The *n*-back task, which has been in use at least since 1958 (Kirchner, 1958), can easily accommodate working load manipulations and different stimulus features. This property allowed it to be used to study WM across various sensory modalities, and a number of early fMRI studies used *n*-back tasks to isolate and compare the neural mechanisms for maintaining and manipulating items in WM. Although not specifically developed to isolate component processes of WM (see Jaeggi et al., 2010), the body of evidence that has developed through the use of the *n*-back task is impressive, not only in the sheer number of published studies but also in what it has revealed about brain regions and networks responsible for WM. In a meta-analytic study, Owen et al. (2005) identified brain regions that are commonly engaged in WM, as assessed by the *n*-back task, and they include: premotor, cingulate, lateral prefrontal, fronto-polar, and medial and lateral posterior parietal cortices. In addition, activations often are observed in perceptual representation-specific regions, including inferior temporal and lateral occipital cortices (Druzgal and D’Esposito, 2001). However, nothing is known about the time course(s) of these brain regions and networks in WM.

In the event-related potential (ERP) literature, Gevins et al. (1996) conducted the first WM ERP studies with the *n*-back task. Using dense-array electroencephalography (dEEG), these authors observed early- (200 ms post-stimulus), mid- (390 ms post-stimulus), and late-latency (600–900 ms post-stimulus) components that differed as a function of WM load. Specifically, early activity was larger for high load conditions, whereas activity at the mid- and late-latency intervals was attenuated for high WM loads.

Using other visual WM paradigms designed to investigate specific WM processes, researchers have shown that activity 300 ms post-stimulus over occipitoparietal recordings sites is sensitive to memory maintenance requirements (e.g., Kiss et al., 1998; McCollough et al., 2007), memory updates (Kiss et al., 1998, 2007), and selection of memory traces for maintenance (Yi and Friedman, 2011). In contrast, frontal components at approximately 400 ms post-stimulus reflect either inhibition of irrelevant stimuli (Yi and Friedman, 2011) or the initiation of updates to the memory store (Kiss et al., 2007).

Studies that employ ERP methodology can leverage both appropriate experimental design and the inherent temporal properties of ERP features to infer functional significance. However, because the vast majority of ERP studies of WM have employed either sparse EEG arrays or analytic techniques such as surface Laplacian or highly simplistic head models that do not take advantage of modern advances in electro-magnetic imaging, information about the relevant underlying brain structures has been lacking. Furthermore, temporal overlap in activity among the underlying neural generators of scalp-recorded EEG can produce complicated scalp voltage distribution patterns that cloud the interpretation of traditionally measured, discrete ERP components (Dien et al., 2004). Consequently, findings from the ERP WM literature have not been integrated with fMRI findings.

Recently, neuroimaging studies have examined the dynamics among the cerebral nodes that support WM by assessing the functional connectivity within and among brain networks during *n*-back performance, leaving an even larger gap between the ERP and fMRI bodies of literature. fMRI functional connectivity studies have observed temporal correlations among the regions noted above during *n*-back performance that are consistent with “task-positive” network (TPN) activity seen in a wide range of tasks (e.g., Fox et al., 2005). Although the specific nodes of this network vary slightly from study to study depending on the task, the method of assessing functional connectivity, and the seed regions used, TPN nodes responsive to *n*-back load typically include lateral prefrontal cortex (lPFC), the insula, the anterior cingulate cortex (ACC), and/or the supramarginal gyrus (SMG – Newton et al., 2011; Gordon et al., 2012; Sala-Llonch et al., 2012). The TPN is believed to support controlled attention processes (Fox et al., 2005), and stronger functional connectivity within the TPN during WM delays is associated with more accurate performance in WM tasks (Pessoa et al., 2002).

Several fMRI studies also have observed functional connectivity in the default mode network (DMN) reflecting task-induced, correlated deactivation among medial frontal and posterior cingulate cortices during *n*-back performance (Hampson et al., 2006; Newton et al., 2011; Gordon et al., 2012; Sala-Llonch et al., 2012). Activity within the DMN is also directly related to task performance. Individual differences in the DMN connectivity strength, whether assessed during a resting state or during task performance, predict individual differences in response accuracy (Hampson et al., 2006; Sala-Llonch et al., 2012).

Thus, while both TPN and DMN functional connectivity are correlated with behavioral performance, the way in which these two networks interact with one another and with other cortical nodes involved in specific aspects of cognition to produce effective task performance remains unclear. Above and beyond the degree to which TPN and DMN connectivity predict task performance individually, the strength of the anti-correlation between these two networks also predicts WM task performance (Hampson et al. (2006)). Furthermore, Chadick and Gazzaley (2011) demonstrated that activity within visual category-specific regions of inferior temporal cortex representing one of two attended stimulus categories (i.e., increased FFA activity when faces were attended and houses were ignored) correlated with activity in the task-positive network. Simultaneously, suppressed activity with regions representing the ignored stimulus category (i.e., decreased PPA activity when houses were ignored) correlated with activity in the default-mode network. While this study clearly demonstrates that the TPN and DMN must coordinate to support task performance in a way that goes beyond simple alternating activity among the two networks, little is known about how these two networks dynamically interact to support performance during a continuous WM task such as the *n*-back. However, understanding the way in which these networks interact to support task performance requires a far better understanding of the temporal dynamics of this interaction than is possible with fMRI.

In the present study, we employed dEEG methodology and an advanced head model for accurate source estimation to examine both the time course and cortical networks involved in WM, as assessed by the *n*-back task. We applied temporal principal components analysis (PCA) and spatial independent components analysis (ICA) to decompose the scalp-recorded brain activity. This analytic approach decomposes signals that overlap in time and space, with each principal component representing cortical activity that is temporally and spatially correlated. Each resulting component is independent from the others. These statistical decomposition techniques also improve source analysis because noise is separated and removed from the signal (Dien, 2012). The results allow us to delineate not only the underlying dynamics of WM networks but also their temporal courses, laying a foundation for future studies that employ more refined paradigms for further isolation of different WM processes.

## MATERIALS AND METHODS

### PARTICIPANTS

Twenty-four right-handed participants from the general student population at the University of Oregon completed the study. Due to EEG data quality (two participants) and behavioral performance (five participants), data from seven participants were not analyzed further. Of the remaining 17 participants, 7 were males, and their age ranged between 19 and 60 (median = 22, SD = 12.4). It is noted that three participants had age ranging from 57 to 60. When these participants were removed (median = 22, SD = 3.3), the significant behavioral and dEEG results reported below do not change. Therefore, we retained these three subjects in all analyses.

All participants had normal or corrected-to-normal vision and reported no history of seizures or loss of consciousness and no current medications or use of illicit drugs that could affect the EEG. The experimental protocol was approved by institutional review boards at EGI, the University of Oregon, and the Office of Naval Research. All subjects provided informed written consent prior and received $30 for participating.

### STIMULI AND EXPERIMENTAL DESIGN

**FIGURE 1:**
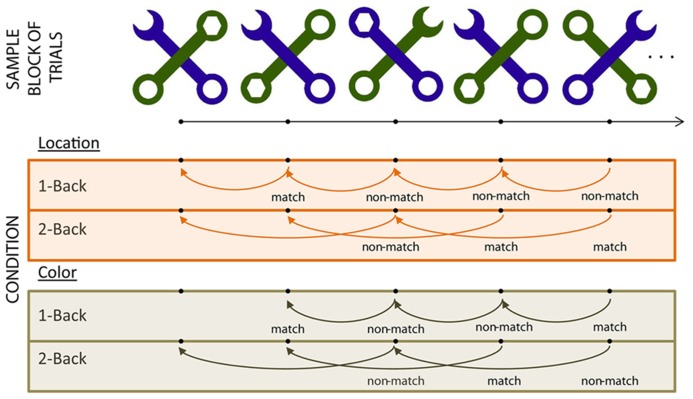
**Task schematic (see Stimuli and Experimental Design)**.

Each stimulus (see **Figure 1**) consisted of two diagonal, overlapping wrenches (Weber et al., 1997; Caggiano and Parasuraman, 2004). In each stimulus, the two wrenches differed in shape (one contained a hexagonally shaped head and the other a C-shaped head) and in color (green or purple). Each stimulus was divided virtually into four quadrants; the head for each wrench appeared in one of these four quadrants in any given stimulus. The total set of stimuli included all possible combinations of wrench color, wrench head quadrant, and depth (which wrench was overlaid on top of the other), resulting in a set of 32 stimuli. Each stimulus from the total available set was used equally often across all experimental blocks. Participants viewed each stimulus at a distance of 65 cm from the monitor, and each stimulus subtended 9.5° × 9.5°.

The experiment employed a 2 (WM load) by 2 (Task – color vs. location) blocked design. Participants indicated whether a key feature of the C-shaped wrench of each stimulus matched the similar feature on the stimulus presented “*n*” trials previously by pressing a button on “match” trials (see **Figure 1**). In the “color” blocks, participants based their comparisons on the color of the C-shaped wrench. In “location” blocks, they based their comparisons on the stimulus quadrant in which the C-shaped wrench head appeared.

On each trial, the stimulus was presented for 2000 ms followed by a randomly jittered inter-stimulus interval of 1000–1500 ms. The experiment included two blocks of each of the WM load × Task conditions, resulting in a total of eight blocks. The order of blocks was randomized with respect to condition with the following constraints – the two blocks for any given condition always were run in succession (i.e., the first 1-back color block was always followed by the second 1-back color block). The study consisted of eight blocks; each block contained 128 trials and ran for approximately 8 min. The condition order was counterbalanced across participants.

### EEG RECORDING

Electroencephalography was acquired using a 256-channel HydroCel Geodesic Sensor Net (EGI, Eugene, OR, USA). All electrode impedances were kept below 70 KΩ (Ferree et al., 2000). Recordings were referenced to Cz. The EEG was low-passed filtered (100 Hz) prior to being sampled at 250 samples/s with a 24-bit analog-to-digital converter.

### PROCEDURE

After providing informed consent, participants were fitted with the 256-channel Hydrocel Geodesic Sensor Net (EGI, Eugene, OR, USA). To minimize head movements, participants rested their head on a chin rest during task performance. Once participants were fitted with the EEG Sensor Net, they received instructions for the first task that they would perform and were given 12 practice trials. In between blocks, participants took a short break, as needed. Participants viewed new instructions and performed 12 new practice trials every time they encountered a block with a new condition. The entire study lasted 3 h.

### EEG PROCESSING

The continuous EEG was band-pass filtered at 0.1–30 Hz with zero-phase shift FIR filters. The data were then segmented relative to the onset of each stimulus (200 ms before and 1000 ms after). Only correctly identified match trials were included in the analysis. Trials were then sorted according to task and load. Trials with blinks or ocular movement artifacts were excluded. Trials with more than 10 bad channels (defined as any sample that exceed a voltage threshold of 200 μV or a sample-to-sample transition threshold of 100 μV) were also excluded. The data were then averaged and re-referenced to the average reference. The data were not baseline corrected prior to submission to the PCA/ICA procedures because we want to examine any WM related effects that precedes stimulus onset, as would be expected for the *n*-back task.

### PRINCIPAL COMPONENTS ANALYSIS

Because the ERPs reflect superimposed activity from multiple sources with overlapping time courses, we decomposed the ERPs using PCA. The average data for each subject were entered into a temporo-spatial PCA using the ERP PCA Toolkit version 2.14 (Dien, 2010, 2012). In this analysis, a temporal decomposition is first performed on the covariance matrix with time points as the variables. The PCA factor structure was rotated using the Promax procedure, with subjects, conditions, and channels as the sources of variance. The temporal components thus reflect patterns of covariance among time points. Following the temporal PCA, spatial decomposition of each time course component was conducted with ICA, wherein channels were now the variables, and the factor structure was rotated to achieve spatial independent components with infomax. Because many components will be generated, our strategy for analysis of the components begins with elimination of all components that do not account for at least 0.5% of the variance. Next, we search for components that are identifiable as ERP components and have been previously reported to be affected by WM load manipulations. These components are tested for statistical reliability using repeated measures ANOVA (see Results section) without correction for multiple comparisons. For the remaining components, they were visually examined to determine if load effects are present. These analyses were performed with Bonferonni correction for multiple comparisons.

### SOURCE ESTIMATES

Each temporospatial component represents multiple sources that functionally covary in time and space. Each PCA/ICA component reflects a static topography, and the only thing that varies is the amplitude of the components over time; analysis of the sources for each component at each time point produces the same source map, with source amplitude changing to reflect changes in the component amplitude. We hypothesize that each component describes activity from either a single brain structure or a network of brain structures that covary together over the task conditions and across individuals. Source estimates were conducted for load-sensitive components derived from the temporospatial PCA/ICA, which are statistically identified.

Sources were estimated using GeoSource (version 2.0) electrical source imaging software (EGI, Eugene, OR, USA). GeoSource uses a finite difference method (FDM) of head tissue conductivity for accurate computation of the lead field in relation to head tissues, where the primary resistive component is the skull. The FDM allows accurate characterization of the cranial orifices, primarily the optical canals and foramen magnum. Tissue compartments of the FDM were constructed from whole head MRI and CT scans of a single subject (Colin27) whose head shape closely matches the Montreal Neurological Institute (MNI) average MRI (MNI305). The MRI and CT images were co-registered prior to segmentation of the brain and cerebral spinal fluid (identified from MRI data), and the skull and scalp (identified from CT images), and the individual’s MRI and CT images were aligned with the cortex volume from the MNI305 atlas with Talaraich registration. The tissue volumes were parceled into 2-mm voxels to form the computational elements of the FDM. Conductivity values used in the FDM model are as follows: 0.25 S/m for brain, 1.8 S/m for cerebral spinal fluid, 0.018 S/m for skull, and 0.44 S/m for scalp (see Ferree et al., 2000). These values reflect recent evidence that the skull-to-brain conductivity ratio is about 1:14 (Ryynanen et al., 2006; Zhang et al., 2006), compared to the 1:80 ratio traditionally assumed.

Source locations were derived from the probabilistic map of the MNI305 average. Based on the probabilistic map, gray matter volume was parceled into 7-mm voxels; each voxel served as a source location with three orthogonal orientation vectors. This resulted in a total of 2,394 source triplets whose anatomic identities were derived through use of a Talaraich demon (Lancaster et al., 2000). Once the head model was constructed, an average of the 256-channel sensor positions was registered to the scalp surface. To compute estimates of the sources, a minimum norm solution with the LORETA constraint (Pascual-Marqui, 2002) was used. All source estimates were performed on the temporospatial components from the grand-averaged data.

## RESULTS

### BEHAVIORAL DATA

Of the 24 participants that completed the study, two participants were excluded from analysis due to excessive ocular artifacts. Data from five additional participants were excluded due to poor performance in at least one task condition. Of these five, three seemed to have confused the response mapping in one of the conditions; the other two were unable to reach a criterion level of performance (75% accuracy).

**FIGURE 2:**
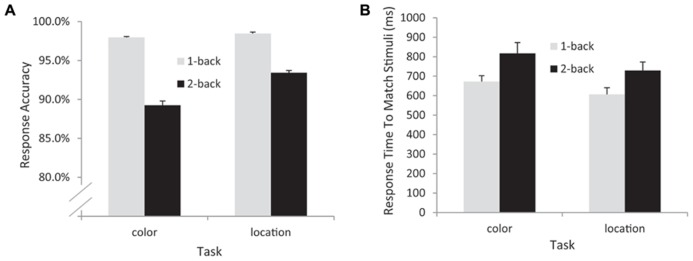
**Behavioral data from all participants included in the EEG analyses.** Accuracy **(A)** and response time **(B)** data. Note that response time data are from correctly detected match trials only.

The response time (RT) and accuracy data from the remaining 17 participants were entered into separate 2 (task: color vs. location) × 2 (load: 1-back vs. 2-back) repeated measures ANOVAs. The RT data indicated significant main effects of task [*F*(1,16) = 9.56, *p* = 0.007, MSE = 10,554] – participants responded more quickly in the location (mean = 668 ms) than in the color (mean = 745 ms) conditions – and WM load [*F*(1,16) = 25.4, *p* < 0.001, MSE = 11,997] – participants were faster in the 1-back (mean = 640 ms) than in the 2-back (mean = 774 ms) conditions. The interaction between task and load was not significant in the RT data [*F*(1,16) = 0.564, *p* = 0.464, MSE = 3609]. The accuracy data also indicated main effects of task [*F*(1,16) = 4.78, *p* = 0.044, MSE = 0.002] and load [*F*(1,16) = 25.4, *p* < 0.001, MSE = 0.003, see **Figure 2**]. Participants responded significantly more accurately in the location [mean_location_ = 95.9%, mean_color_ = 93.6%] and in the 1-back conditions (mean_1-back_ = 98.2%, mean_2-back_ = 91.3%). In addition, a significant task × load interaction [*F*(1,16) = 5.84, *p* = 0.028, MSE = 0.001] indicated that the effect of load was larger for the color task (an 8.7% difference in accuracy) than for the location task (a 5% difference in accuracy, see **Figure 2**).

### EVENT-RELATED POTENTIALS

**FIGURE 3:**
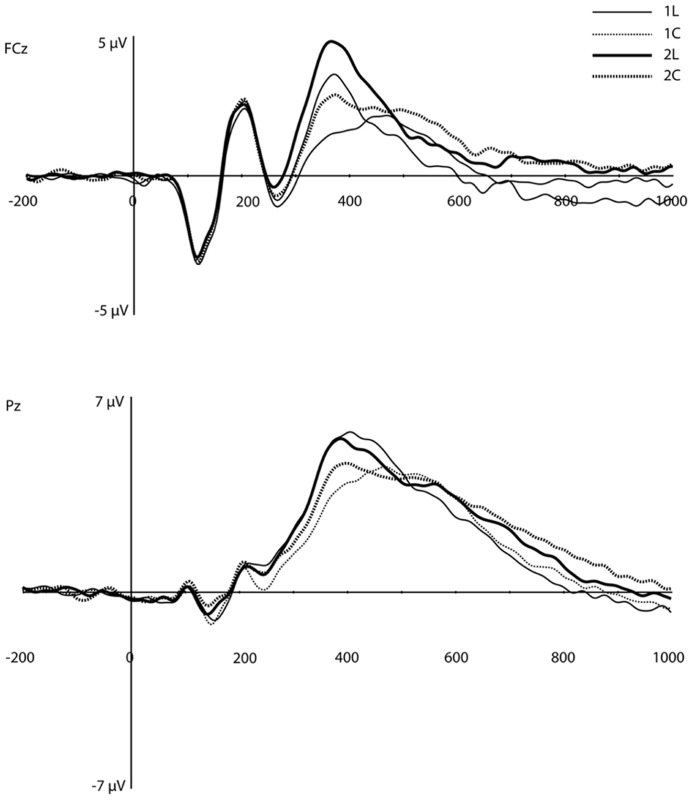
**Original grand-average ERP waveforms at FCz and Pz.** Note that in this figure, a baseline correction (between -200 ms and stimulus onset) was applied, in contrast to all PCA/ICA components displayed in **Figures 4–6**.

On visual inspection, several traditional ERP components appeared to be sensitive to WM load (**Figure 3**). At mediofrontal sites (at ~350 ms), a positive component was larger for the 2-back than the 1-back location condition. At approximately 400 ms over centroparietal sites, the late positive complex (LPC; i.e., P3) also differentiated between WM load, with 1-back location showing the largest amplitude (see Introduction, especially for the location task). Following the peak of the LPC, slow negative-going features of the ERP over frontal and posterior sites also differed as a function of load.

#### Principal components analysis

Temporal PCA decomposed the traditional ERP data into distinct temporal factors. A parallel test (Horn, 1965) was performed to determine the number of temporal factors to retain for the spatial decomposition step. In this test, a Scree plot is generated for a fully random dataset (with the same dimensionality as the actual data set) for comparison with the Scree plot from the actual data. The point at which the two Scree plots cross indicates the number of factors to be retained. Based on the parallel test, eight factors, accounting for 93% of the variance, were retained. A second parallel test on the spatial ICA of the eight temporal factors indicated that four spatial factors, accounting for 78% of the total variance, should be retained for each temporal factor.

Of the 32 temporospatial factors recovered, we eliminated 21 components because they each accounted for less than 0.5% of the variance. Thus, we are left with 11 components for analysis. Applying our criterion of component resemblance to known ERP components (with regards to latency and scalp distribution) that have been shown in previous research to be affected by WM load, we were able to identify two components [Component 1 (C1) and Component 9 (C9), see below]. After this step 9 components remained for exploratory analysis, wherein Bonferroni correction (at 0.05) was applied.

Repeated measures ANOVAs were performed on the scalp voltage data at the peak channels and latency for each component, with Task and Load as within-subjects factors. Of the 11 components, three components showed statistically reliable differences in WM load. We present the analysis of each of these temporospatial components in the following sections.

#### Component 1 (C1)

**FIGURE 4:**
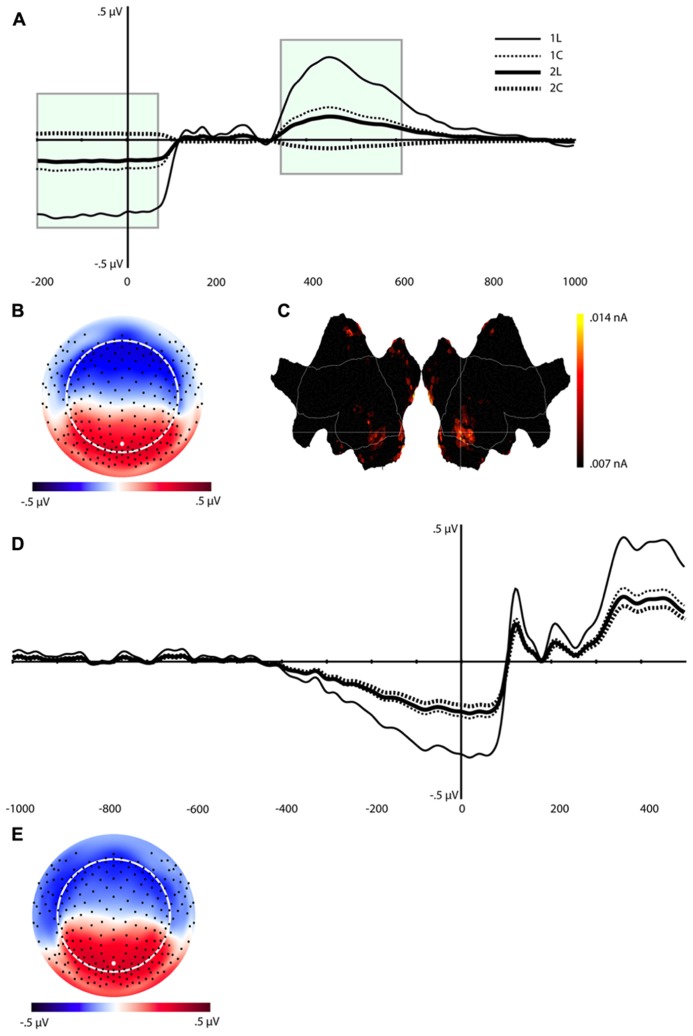
**Component (C1). (A)** Component waveform. Green boxes represent the time windows used for statistical analysis. **(B)** Topographic map showing voltage distribution of C1. Orientation is top looking down with nose at the top. Positive voltage values are red and negative values are blue, with the zero crossing in white. Dots on the map are channel locations and white dot represents channel used for the waveform in **(A)**. **(C)** Source results projected onto a schematic flat map (unfolded cortex) to show activity at all cortical sites. The area within the white border represents the lateral surface (outside of this is the medial wall); the left side is the left hemisphere. Activity was thresholded to show the top 10% of source activity. **(D)** This component waveform was re-computed with temporospatial analysis using a long prestimulus interval [see Component 1 (C1)]. **(E)** Topographic map showing voltage distribution, with the same topography as when computed with a shorter baseline. This component also showed a similar pattern of cortical source activity as the C1 in **Figure 4C**.

The waveforms and scalp topography of C1 are illustrated in **Figure 4**. In this figure, the grand-average ERP waveforms (**Figure 4A**) are presented for all four conditions at the channel overlaying the location indicated in the topographic map (white dot in **Figure 4B**). C1 is characterized by load effects in both the pre-stimulus interval and in a post-stimulus interval that peaks at 456 ms. This component is similar to the LPC, which includes the P3 and related components (see Dien et al., 2004), in its topography and time course in the post-stimulus interval. This same temporospatial component, however, also seems to capture the pre-stimulus stimulus preceding negativity (SPN). Statistical tests were performed on two windows, defined through visual inspection, of C1 (-200 to 80 ms, and 340–630 ms, highlighted in green in **Figure 4A**). We derived an average of each window re-referenced to the 130–300 ms post-stimulus interval (where differences between the four waveforms were minimal). The data were then submitted to repeated-measures ANOVA with Task, Load, and Time (Pre-stimulus vs. Post-stimulus) as within-subjects factors. Note that because this component reflects the LPC/SPN components that have been noted to be affected by load manipulations (see Discussion), significance level was not adjusted for multiple comparisons.

The ANOVA revealed significant effects for Load, *F*(1,16) = 8.4, *p* < 0.02, Task, *F*(1,16) = 5.3, *p* < 0.04, Time × Load, *F*(1,16) = 8.4, *p* < 0.02, and Time × Task (1,16) = 5.3, *p* < 0.04 (see **Table 1**). The Time × Load effect indicated that although the amplitude was larger in the 1-back than in the 2-back condition in both time windows, the polarity of the component reversed from the pre-stimulus to the post-stimulus window. Paired *t*-tests performed at each time interval revealed that the differences were significant – *t*(16) = -2.9, *p* < 0.02 (pre-stimulus), *t*(16) = 2.9, *p* < 0.02 (post-stimulus). Similarly, inspection of the mean for the Time × Task effect showed that the location task was associated with greater amplitude overall, but the polarity of the component differed over time. Paired *t*-tests also confirmed that during the pre-stimulus interval the location task was associated with greater negative amplitudes than the color task, *t*(16) = -2.3, *p* < 0.04, whereas the location task elicited larger positive amplitudes than the color task during the post-stimulus interval, *t*(16) = 2.3, *p* < 0.04.

**Table 1:** Summary table of components and experimental effects.

Component	Source location	Talairach coordinates of maximum current density	Effect	Significance
C1	Bilateral inf. temp. lobe, BA 18, BA 7, PCC	60, -53, -13	Load	*p* < 0.02
			Task	*p* < 0.04
			Time	Ns
			Time × load	*p* < 0.02
			Time × task	*p* < 0.02
C7	Bilateral inf. temp. lobe, bilateral temp. poles, BA 18, BA 10, BA 11, BA 46	-3, 45, -20	Load	*p* < 0.002 [-1pc]
			Task	Ns
			Time	*p* < 0.001
			Time × load	*p* < 0.002
			Time × task	Ns
			Task × load	Ns
			Time × task × load	Ns
C9	Bilateral inf. temp. lobe, bilateral temp. poles, BA 18, middle and superior temporal gyri, BA 10, BA 11, BA 40	60, -53, -13	Load	*p* < 0.002 [-2pc]
			Task	*p* < 0.002
			Load × task	Ns

*PCC, posterior cingulate cortex.*

In order to understand the pre-stimulus time course of C1 over a somewhat longer interval, we re-segmented the data with a window of 1000 ms (vs. the initial 200 ms) before and 500 post-stimulus onset. Because more subjects blinked in the longer pre-stimulus interval, the number of participants with enough artifact free trials for signal averaging was reduced from 17 to 14.

The same temporospatial PCA/ICA procedure was applied to this subset of subjects, and the parallel test revealed that 10 temporal factors, accounting for 94% of the total variance, should be retained for the next (spatial ICA) step. The parallel test for the spatial ICA step revealed four spatial components should be retained for each temporal component, accounting for 81% of the total variance. Examination of these components revealed a component with a similar time course, topography, and condition differences as C1 (**Figures 4D,E**). Consistent with the interpretation of an SPN, C1 activity in this longer interval began to deviate from baseline approximately 400 ms before stimulus onset (**Figure 4D**).

**FIGURE 5:**
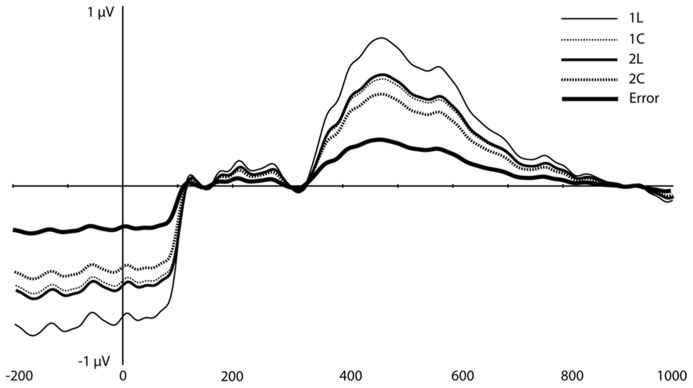
**Component 1 (C1) waveforms with error trials**.

In considering the pre-stimulus activity for the C1 component, if this activity is in fact an SPN, reflecting functional engagement of visual association in preparation for the visual perception, then trials with a smaller C1 or SPN would be expected to have lower accuracy. The Temporospatial (PCA/ICA) analysis was re-run for the 11 subjects with enough errors to include an average for error trials. **Figure 5** illustrates that the C1 component amplitudes for the correct trials show a similar pattern as for the full sample. As predicted, the C1 amplitudes are markedly attenuated for error trials, consistent with the interpretation that the pre-stimulus negativity of C1 is a functional preparation for effective visual perception.

Source estimation for the grand-average of C1 showed prominent activity in the bilateral inferior temporal lobe (including the fusiform area), BA 18, and BA 7 (precuneus), and posterior cingulate cortex (PCC, see **Figure 4C**). The inferior temporal lobe activity was bilateral but stronger for the right hemisphere.

#### Component 7 (C7)

**FIGURE 6:**
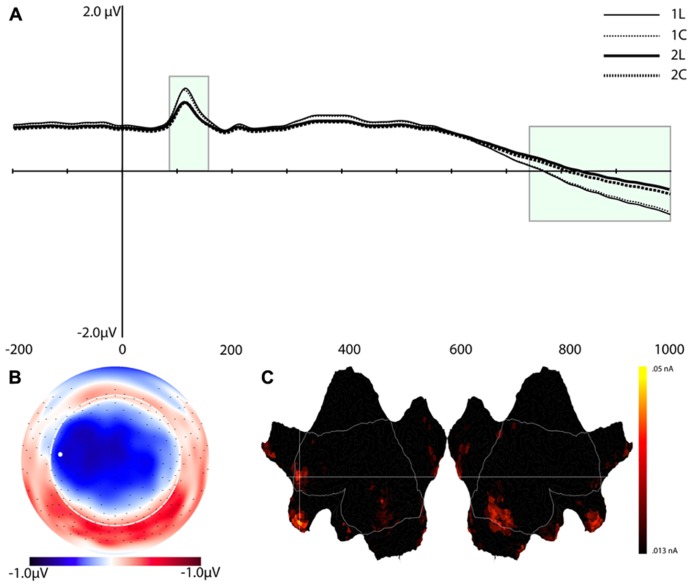
**Component 7 (C7).** **(A–C)** are as in **Figure 4**.

**Figure 6** shows the waveforms and spatial topography of C7. This component also had two post-stimulus time intervals, one early (88–156 ms) and one late (700+ ms), that were sensitive to WM load. We analyzed this component using a repeated measures ANOVA with Task, Load, and Time (Interval 1, Interval 2) as within-subjects factors, referenced to the average of the -200 to 0 ms prestimulus interval. The adjusted significance level for multiple comparison is *p* < 0.005. The main effects of Load, *F*(1,16) = 18.2, *p* < 0.002, and Time, *F*(1,16) = 37.7, *p* < 0.001, were significant, as was the Load × Time interaction, *F*(1,16) = 18.2, *p* < 0.002 (see **Table 1**). Paired *t*-tests showed that at both time intervals the waveforms differed significantly, *t*(16) = -2.3, *p* < 0.04 (early), *t*(16) = 2.3, *p* < 0.04 (late).

Source estimation results showed that, in addition to the extrastriate areas that we observed for C1, C7 included activation in the left middle frontal gyrus (MFG, BA 10), left inferior frontal gyrus (IFG, BA 46), medial orbitofrontal cortex (OFC, peak at BA 11), and temporal poles (stronger for the right hemisphere, see **Figure 6**).

#### Component 9 (C9)

**FIGURE 7:**
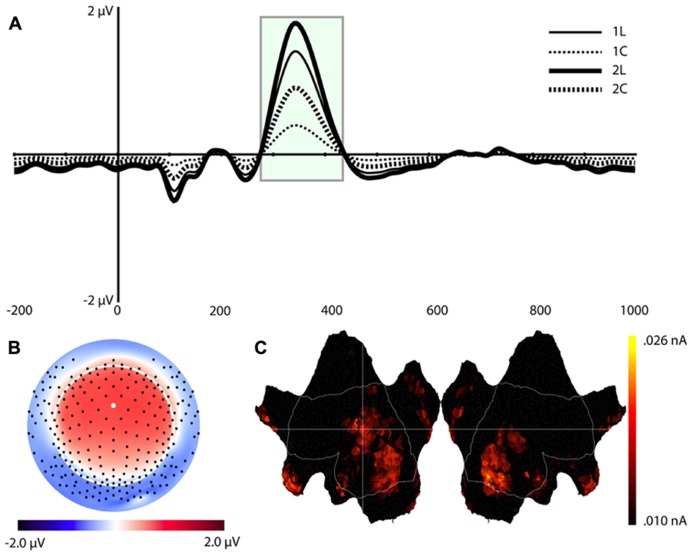
**Component 9 (C9).** For each component, **(A–C)** are as in **Figure 4**.

This component was most prominent at approximately 350 ms post-stimulus (see **Figure 7**, C9) and resembles the P2 component and is affected by the load manipulation as previously reported (see Discussion). C9 was quantified as the mean activity 280–430 ms post-stimulus and referenced to the mean of the 200 ms pre-stimulus interval. Analysis included Task and Load factors. The main effects were significant – Task, *F*(1,16) = 18.2, *p* < 0.002, and Load, *F*(1,16) = 15.7, *p* < 0.002 – confirming that the amplitude was largest in the 2-back and in the location conditions (see **Table 1**).

Source estimates were similar to C1. In fact, the location with maximal current density is the same as C1 (see **Table 1**). However, C9 also included activity in the middle and superior temporal gyri and in the inferior parietal lobe (BA 40, stronger in the left hemisphere, **Figure 7C**, C9) not present in C1. C9 also had sources that were similar to C7, notably activity at the temporal poles and medial prefrontal cortex.

#### Network dynamics

**FIGURE 8:**
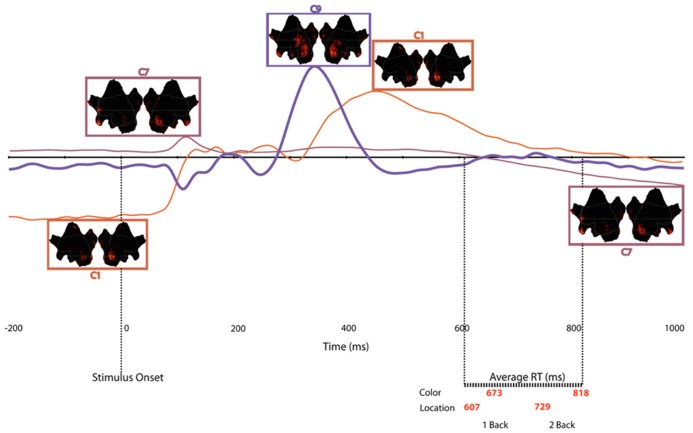
**Time course of each temporospatial component in relation to stimulus onset and mean reaction times for each experimental condition.** Note that border of source maps are color coded to match waveforms.

Together, the three components described above capture the essential aspects, both in terms of neural regions, as well as in time course of activity, of an overarching network that supports WM in the *n*-back test. To illustrate the sequence of component activations, **Figure 8** plots the time courses, with insets of cortical activation patterns, for all of the components that varied significantly as a function of memory load. The underlying dynamics of this network seem to follow this time course:

(a) Engage active control (C1), 200 ms before stimulus onset to 80 ms post-onset
(b) Prime attentional resources (C7), 88 ms post-stimulus onset to 156 ms post-onset
(c) Focus attentional resources (C9), 280 ms post-stimulus onset to 430 ms post-onset
(d) Release control (C1) 340–630 ms post-stimulus onset
(e) Re-prime for the next trial (C7), 700 ms post-onset

## DISCUSSION

In this *n*-back WM task, the subjects attended to a relevant feature of the visual stimulus (color or location), held it in memory, and evaluated whether that feature matches the same feature category on either the next trial (1-back) or the trial after the next trial (2-back). RT analysis indicated that, whereas the color memory task was more difficult overall, the effect of the WM load manipulation was roughly equivalent for the color and location tasks. While the accuracy data showed a significant interaction between task type and task load, with a larger effect of load in the color task, this interaction was likely due to a combination of the main effect of task with a ceiling effect in the 1-back conditions – participants were at or near ceiling in terms of accuracy for 1-back for both the color (98% correct) and the location (98.4% correct) conditions. Nonetheless, RT was faster for the location 1-back condition (mean RT = 607 ms) than the color 1-back condition (mean RT = 673 ms; *F*(1,16) = 8.46, *p* < 0.01).

Having validated the WM load manipulation in the behavioral data, we then examined the brain’s electrical activity that varied with WM load. To separate the brain activity superimposed at the head surface from multiple cortical sources, we conducted a temporospatial analysis, in which a temporal PCA was followed by a spatial ICA. The temporal PCA identified unique time courses (temporal components) that suggested patterns of covariance in time computed over the spatial features of brain activity (variability over channels), the task demands (variability over conditions), and individual differences (variability over subjects). The subsequent spatial ICA decomposition showed only three temporospatial component that varied with memory load. Our reasoning was that the temporospatial decomposition – when examined in relation to WM load – may suggest how to *statistically* decompose the complex time course of cortical activity into functionally meaningful patterns (i.e., networks). In some cases, these patterns may indicate activity from a single, modular brain source, while in other cases, these patterns may indicate networks of cortical function that must be coordinated to achieve effective WM. The LORETA source estimation maps provide a data-driven approach that makes few assumptions regarding modular vs. distributed network accounts of temporospatial ERP components. We first discuss the possible meaning of each temporospatial component, and then consider them together as an ensemble of interacting networks.

### TIME COURSES AND SPATIAL PATTERNS OF CORTICAL ACTIVITY IN WORKING MEMORY

#### Component C1

Component C1 peaked at 456 ms post-stimulus, similar to the LPC. The memory load effect for C1 replicates load effects for the LPC reported by other researchers (Gevins et al., 1996; McEvoy et al., 1998). PCA studies show that the LPC can be decomposed into several components with unique topographies and time courses (Dien et al., 2004; Foti et al., 2007). One component identified by Foti et al. (2007) has an occipital distribution and a time course that seem to parallel C1. The apparent network pattern of C1, including both visual association areas and a more general (PCC) limbic cortex control function, is consistent with a major component of the LPC.

However, the C1 component in the present study also showed a pre-stimulus negativity that exactly mirrored the post-stimulus positivity of the apparent LPC starting at 350–400 ms. This pre-stimulus negativity is not typically attributed to the LPC in the literature. This may be due to the fact that for conventional ERP analysis, the typical practice of pre-stimulus baseline correction may mask the relationship between any pre-stimulus negativity and the LPC response. In the early literature on the P300 (a component closely related to the LPC), there was a controversy over whether the P300 could be interpreted as a “release” of the contingent negative variation (CNV). The CNV is a pre-stimulus negativity that appears to reflect preparation for processing, and perhaps for responding to, the stimulus (Williamson et al., 1977). Although Donchin and Heffley (1979) used factor analytic evidence to argue persuasively that the P300 and the CNV are distinct components, the present findings suggest that pre-stimulus negativity may figure importantly in P300-like activity under certain task demands, such as placing explicit demands on WM. The pre-stimulus load effects on C1 are consistent with prior studies with the *n*-back task. McEvoy et al. (1998) observed WM load effects on CNV, and the time course of these effects was similar to that of the C1 component in the present study.

The CNV, initially described as the E wave or expectancy wave (Williamson et al., 1977), was later decomposed into two components: an early component reflecting an orienting response, and a late motor preparation response. More recent findings show that the late component of the CNV can be decomposed further into those components that reflect motor preparation and those that reflect anticipation of the arrival of sensory stimuli (Brunia and van Boxtel, 2001). The expectation for the sensory perception is most relevant to the present C1, which showed pre-stimulus negativity over visual association areas. This perceptual readiness component has been described as the SPN. Brunia and van Boxtel, 2001 presented evidence that the SPN reflects anticipatory attention that elicits sensory-specific cortical activation and reasoned on neuroanatomical grounds that the SPN, although generated in cortex, reflects control influences from the pulvinar nucleus of the thalamus. In the current study, C1 was generated in visual cortices (particularly on the right side) and in PCC, consistent with prior research showing that sources of the late CNV interval seem to engage the PCC, lateral and medial occipital cortex, and frontal regions (Gómez et al., 2003). Somewhat in contrast with prior work, however, C1 was separated from frontal regions, and the PCC was the apparent limbic control point for maintaining the priming in visual association cortices. Locally, the functional priming of visual cortex may be achieved through modification of the cortical depolarization threshold, manifesting as a surface negative potential (Rockstroh et al., 1993). Such an effect may be achieved through subcortical regulatory input from the pulvinar nucleus of the thalamus (Brunia and van Boxtel, 2001).

It may seem counterintuitive that the apparent priming of visual cortex was greater for the low load than the high load condition. A similar effect was also observed by McEvoy et al. (1998). This effect might indicate that for the low load condition the perceptual representation (and priming negativity or SPN) is maintained into the next trial, whereas for the high load condition the target perceptual representation must be re-accessed and is not maintained in the pre-stimulus interval.

Results from fMRI research are consistent with this interpretation that there is local priming of visual cortex when the representation of a visual stimulus is held in a perceptual store. Kastner et al. (1999) showed that when attention was allocated to a location that would eventually contain a visual stimulus, the visual cortices showed increased activity during the expectant period, even when no stimulus was present. Esterman and Yantis (2010) observed similar effects within object-selective regions of temporal cortex when participants were cued to anticipate a particular category of visual stimulus. Consistent with the interpretation that a greater priming negativity (SPN) reflects greater anticipatory attention, the amplitude of C1 in the present study varied with RT, with larger C1s and faster responses not only for the low load vs. high load, but also for the location vs. color conditions.

A similar reasoning can be applied to the post-stimulus interval, where C1 peaked at 456 ms and mirrored the distribution of amplitudes over experimental conditions seen for the pre-stimulus interval. The response of C1 in the LPC interval was inversely related to the level of pre-stimulus priming. Although the C1 response in the LPC interval may reflect an active control mechanism, this activity might also reflect a kind of releasing mechanism, wherein the activity of the visual cortex is released from the control of attention circuits (such as the pulvinar, or perhaps the prefrontal cortex or temporoparietal junction – TPJ).

It is important to note that the C1 response is not an early visual response such as indexed by the P1 or N1. Those components were not affected by load, either in the present study or in previous studies (McCollough et al., 2007). Rather, the post-stimulus C1 is a late response in visual association cortices and in memory-related limbic structures (PCC). Furthermore, the apparent network indexed by the C1 is not the classical P3b network – the C1 topography differs substantially from the traditional P3b topography (see Foti et al. (2007), even though it does share the PCC as a pivotal source with the P3b (see Luu et al., 2007).

Considering both time windows of the C1, this component seems to reflect a functional priming and then functional activation of the visual perceptual network. If so, then we would expect an attenuated C1 (poor expectant priming) on trials with errors. In fact, a weak pre-stimulus C1 should predict a high likelihood of an error on that trial. **Figure 5** shows the data for subjects with sufficient errors to create ERP averages for errors as well as for correct responses. The pattern of results for correct trials is similar to the analysis of the full sample. Furthermore, as predicted there is an attenuated C1 component for error trials.

#### Component C7

This component (**Figure 6**) reflects a significant enhancement for the low load vs. high load conditions early in the post-stimulus epoch, then apparently releasing slowly. To our knowledge this component has not been reported in the literature. Source analysis included right-lateralized activity in visual association cortex, similar to the area primed by C1, as well as right-lateralized temporal pole sources, perhaps consistent with the spatial demands of the task. In fMRI studies, temporal pole activation has been related to holistic perception of objects (Zhou et al., 2010). Furthermore, multiple structures of the anterior temporal lobe (superior, middle, and inferior temporal gyrus, perirhinal and entorhinal cortices, and uncus) have been shown to form a functional network involved in declarative memory processes (Gour et al., 2011).

Whereas these sources seem clearly relevant to the right-lateralized processing of the figural stimuli of this task, C7 was strongly *left-*lateralized for the sources in the frontopolar (MFG, IFG, and OFC) regions. The MFG and IFG have been identified in previous research as integral to a stimulus-driven ventral attention network (Corbetta et al., 2008). According to Corbetta et al. (2008), the ventral attention network is engaged by the appearance of important stimuli, independent of the salience of the stimuli. Specialized for object attention and memory, the ventral attention network seems to be particularly well developed in the left hemisphere, which supports analytic perception and object memory (Tucker et al., 2007; Tucker and Luu, 2012). Engagement of the OFC may be expected for the ventral attention network as well, particularly as it contributes to WM performance. Recent evidence shows that lesions to the OFC impair coordination of multiple discrete cognitive processes in the *n*-back task, such as simultaneous maintenance, manipulation, and monitoring of information (Barbey et al., 2011).

Based on fMRI studies, the control of the (right-lateralized) visual association areas (such as seen in C7) might be expected to engage the frontal networks of the (left-lateralized) ventral attention system (also seen in C7). However, the time course of this C7 component is remarkably early, with an abrupt peak at ~100 ms as the earliest feature of the visual ERP in this study. On the surface, our current results, suggesting that frontal networks are engaged early, runs counter to the ERP literature, which shows that such early neuronal responses are typically observed over sensory cortex. How could frontal networks be engaged so early?

One possibility is that frontal attention control is engaged through non-specific thalamic projections in parallel with the sensory-specific thalamic projections to primary visual cortex. The frontal pole (reflected in the activity of C7) is particularly important to the thalamic regulation of attention, suggesting the possibility of two-way frontothalamic interactions in the early stages of attentional control. The frontal polar cortex in primates is connected to the anterior thalamic reticular nucleus (TRN, Zikopoulos and Barbas, 2006), which regulates thalamocortical activity (including sensory traffic). This anatomical organization puts the TRN, and its frontopolar cortical network, in a central position to regulate selective attention processes (Yingling and Skinner, 1997), as well as the general state of alertness (Tucker et al., 2007). A now classic hypothesis (Crick, 1994) proposes that the TRN’s inhibitory control over thalamocortical projections provides the neural mechanism for the spotlight of attention. Furthermore, recent evidence shows that seizure discharges in the frontal pole are observed in absence spells with a momentary lapse in conscious attention (Tucker et al., 2007). In support of this proposal, early post-stimulus attentional selection-related processing has been observed in monkey PFC (Hasegawa et al., 2000; Everling et al., 2002).

Whatever the neural mechanism, the time course of C7 suggests that the component is re-engaged late in the epoch (**Figure 6**). That this activity returns after a response is made may suggest that there is attention modulation of the same areas in anticipation of the next trial, given the evaluation of present stimulus in the context of the 1-back or 2-back memory requirements.

#### Component C9

Previous ERP research has produced somewhat contradictory results on WM load effects within this time window. Gevins et al. (1996) showed that a vertex-positive component occurring at approximately 200 ms post-stimulus was larger in high-load than in low load conditions for matching stimuli. In contrast, McEvoy et al. (1998) showed no effect of load for matching stimuli in the vertex P250, but P250 was larger for a spatial task compared to a verbal task.

At the scalp, C9 in the present study showed a peak positivity near the vertex (see **Figure 7**) at roughly the same time as the P200/P250 previously reported (Gevins et al., 1996; McEvoy et al., 1998). Like the P200/P250, the present C9 showed effects both of load (larger for high load, as reported by Gevins et al., 1996) and of task (larger for location compared to color, as reported by McEvoy et al., 1998). The conflicting results of load and task effects found by Gevins et al. (1996) and McEvoy et al. (1998) might be due to differences in experimental procedures, the use of different stimuli, quantification errors of the P200/P250 because of temporal overlap of different underlying components, or the use of surface Laplacian measures that are only sensitive to superficial cortical sources. In our analysis, the PCA/ICA procedure statistically separated C9 from other overlapping components, making its quantification more discrete.

We consider C9 to reflect the activity, engaged strongly at this time interval, of the ventral network identified by Corbetta et al. (2008). Whether functionally separable or not, these components reflect the importance of the posterior aspect of the superior temporal gyrus, occipital cortex, and the inferior parietal lobe in the control of attention in this WM task.

As noted by Corbetta et al. (2008), previous research has shown that activity in the TPJ is *deactivated *when participants have to keep more information in mind. This effect was interpreted as reflecting the suppression (by a dorsal, goal/task oriented network) of the reorienting network to enable memory traces to be maintained during a retention interval. Consistent with this reasoning, they noted that during high-workload conditions, novel unattended stimuli are less likely to be detected. Chambers et al. (2004) also showed, using transcranial magnetic stimulation, that when the right TPJ is stimulated between 90–120 ms and 210–240 ms after stimulus onset, reorienting to a new stimulus is impaired. They argued that the later effect reflects a different stage of processing within the slower visual pathway. The slower time course identified by Chambers et al. would place that effect at about the onset of the present C9 component.

### TIME COURSE OF MULTIPLE NETWORKS IN WORKING MEMORY

Considering the multiple load-dependent components together, the temporospatial analysis revealed networks (statistically defined by PCA and ICA) that span a pre-stimulus preparation to post-response consolidation interval (**Figure 8**). Across time, three networks emerged, with each network sharing some common cortical nodes and, critically, involving unique, non-shared nodes as well (see **Table 1**). The first network to appear in the data involves bilateral visual association areas and the PCC. The pre-stimulus negativity of C1 seems to reflect priming of visual cortex for cognitive processing of the trial through an anticipatory attention mechanism. Consistent with this notion, fMRI studies have shown that the PCC is part of the DMN and that greater PCC *deactivation *is associated with faster reaction times (Chadick and Gazzaley, 2011). Similarly, Anticevic et al. (2010) showed that, compared to error responses, correct responses were associated with greater *deactivation *of the DMN. In the present study, although we have considered the PCC as providing executive control over visual priming (Mesulam, 1981), it might be important to consider the alternative that the PCC element of the pre-stimulus C1 may reflect *suppression *of the PCC and thus DMN in order to support the perceptual priming or memory maintenance required for task performance.

Shortly after stimulus onset, the C1 network appears to be released, and then at about 100 ms there is abrupt activity in all other time course components that reflect memory load effects. One of these components (C7) shows a brief peak at about the same time as C1 activity diminishes and then goes quiescent until a slow opposing ramp late in the epoch. The cortical network engaged by C7 seems to reflect recruitment of attentional control (left frontopolar), holistic memory representation (right anterior temporal), and priming of visual association areas. Even though it is engaged just briefly, we speculate that this C7 network sets the stage, through top-down control, for further processing.

It is important to point out familiar ERP components, P1 and N1, which are missing from **Figure 8** because they do *not *vary with WM load. Some later scalp topographic features of the conventional N1 may be discerned in the map of the C9 component (**Figure 7**), but these are posterior negative inversions of the familiar frontal P2 reflected by C9, and are not reflective of the primary N1 component. Thus, although some of the sources of activity noted across the various components reported here bear similarity to cortical nodes activated during selective attention tasks, the temporal effects of WM load on neural processes appear to occur later than the earliest effects reported for spatial (Martinez et al., 1999) and color-based (Zhang and Luck, 2008) attentional selection tasks.

In the conventional P2 and early P3 interval of the ERP (280–430 ms), a set of networks show load effects. C9 reflects the familiar anterior centrofrontal P2 of the ERP, and one interpretation is that it re-engages networks previously primed or activated by the C1 and C7 components. Certain structures disappeared (such as the lateral MFG and IFG seen in C7) and new ones were added (such as the TPJ in C9). This P2/C9 network may reflect attentional modulation, through regulation of orienting responses to the presented stimulus, and it involves not only sensory areas but also the OFC, which is active when attention must reorient following invalid cues (Nobre et al., 1999) and the temporal poles. After the 280–430 ms interval, the C1 network reappears to contribute to the later LPC (Dien et al., 2004; Foti et al., 2007). Finally, C7 ramps in an opposite direction to its initial deflection late in the epoch after responses have been made. Perhaps this suggests that the same processes involved in preparing processing for the stimulus on a current trial are re-engaged to prepare processing on the next trial.

With the exception of the C1 component, the cortical regions engaged by C7 and C9 reflect temporospatial networks of the ventral attention network identified by Corbetta et al. (2008). The temporal resolution of dEEG allowed us to delineate how the component networks of the ventral attention system, such as the MFG, IFG, and TPJ, emerge at different points in time. Consistent with the reactive (stimulus-initiated) nature of the ventral attention system, C7 and C9 were reactive in the post-stimulus interval.

### LIMITATIONS OF THE PRESENT STUDY

This study was designed to manipulate WM load parametrically, through the *n*-back task. However, the conventional cognitive psychology interpretation of this task, as a memory process that must be held in an active store for a short time or a longer time, may be incorrect. Particularly when observing the dEEG evidence that there was priming of visual association areas, and that this was most strong for the 1-back condition, it seems apparent that the 1-back condition is a different cognitive task, which involves holding a percept in an active visual store, than the 2-back condition, which does not involve maintenance of a perceptual store (which is impossible because of the intervening stimulus) but rather requires a different (perhaps more abstract or symbolic) access of the 2-back stimulus. This finding could also be accommodated by an account grounded in dual-process theories of memory, in which declarative and non-declarative representational systems contribute in varying proportions to *n*-back performance and in which declarative processes play a greater role under 1-back conditions than under 2-back conditions (Caggiano et al., 2006; Vilberg and Rugg, 2008). Further research is required to determine if the difference between the 1-back and 2-back conditions is indeed qualitative, as seems in the present study, rather than a quantitative difference in memory load.

Although the *n*-back task is commonly used to study WM processes, the component processes (such as storage, maintenance, and executive control) are not readily separable. Our interpretation of the functional significance of each component not only relied on the task effects and time course, but also previous findings. *Therefore, our interpretations must be tested in future research, using paradigms that can separate different WM processes, such as storage, rehearsal, and executive processes* (e.g., Kiss et al., 2007).

The use of the WM load manipulation to identify functionally relevant components in the temporospatial analysis caused us to select certain components from the larger set of temporospatial components that are important to understand the cognitive processes common to the two memory load conditions. Future research should not only examine which of the present components replicate clearly, but also examine these in light of the complete set of time courses and cerebral networks required for all elements of task performance.

## CONCLUSION

The present study suggests that a temporospatial analysis can separate several unique time courses of neural activity with different underlying cortical networks that contribute to WM performance. In recent cognitive models, WM is considered to be served by top-down, temporary activation of items in long-term memory (Ruchkin et al., 2003) via directed attention (e.g., Cowan, 1997; Postle, 2006). The present results suggest that temporospatial analysis of the ERP can isolate the contribution of key nodes in the frontal lobe, TPJ, and PCC that appear to mediate directed attention. Nonetheless, the cortical activity most clearly related to this visual WM task was not an abstract representation from long-term memory, but the priming of visual cortex that seemed to reflect preparation for a direct match between the representational expectancy and the visual percept.

## AUTHOR CONTRIBUTIONS

Phan Luu, Daniel Caggiano, Alexandra Geyer, and Jenn Lewis contributed to the design of the study. Jenn Lewis was responsible for data acquisition. Phan Luu, Daniel Caggiano, Alexandra Geyer, Jenn Lewis, and Don Tucker contributed to data analysis. All authors contributed to the interpretation and preparation of the manuscript. All authors approve the version that is currently under consideration and acknowledge that they are accountable for all aspects the work.

## Conflict of Interest Statement

The authors declare that the research was conducted in the absence of any commercial or financial relationships that could be construed as a potential conflict of interest.
